# The complete mitogenome of *Montipora digitata* (Cnidarian: Acroporidae)

**DOI:** 10.1080/23802359.2018.1450656

**Published:** 2018-07-25

**Authors:** Xinming Liu, Chenying Wang, Xiaofeng Shi, Xinqing Zheng

**Affiliations:** a Third Institute of Oceanography, State Oceanic Administration, Xiamen, China;; b Institute of Oceanology, Chinese Academy of Science, Qingdao, China;; c Guangxi Academy of Oceanography, Nanning, China;; d College of Ocean and Earth Sciences, Xiamen University, Xiamen, China

**Keywords:** *Montipora digitata*, mitogenome, next generation sequencing

## Abstract

In this study, the complete mitogenome sequence of *Montipora digitata* (Cnidarian: Acroporidae) has been decoded for the first time using the low-coverage whole-genome sequencing method. The overall base composition of *M. digitata* mitogenome for A, C, G, and T is 24.9%, 14.2%, 24.2%, and 36.8%, respectively, with a GC content of 38.3%. The assembled mitogenome consists of 17,884 bp with unique 13 unique protein-coding genes (PCGs), two ribosomal RNAs, and only three transfer RNAs genes. *M. digitata* has one big intron insertion in the ND5 gene. The complete *M. digitata* mitogenome provides essential and important molecular DNA data for further phylogenetic and evolutionary stony coral analysis.

*Montipora digitata* belongs to *Cnidiria* phylum, *Anthozoa* class, *Scleractionia* order, and *Acroporidae* family (Sims 2007). This species are widely distributed throughout the Indo-Pacific Ocean from East Africa to the Marshall Islands and Fiji (Dai and Horng 2009). The colonies are always found in shallow habitats (Veron 1995). It can be a dominant species of shallow mud reef flats. It was also found to be a pioneer species of reef flats during natural reef recovery after catastrophic destruction (Li et al. 2015). *Montipora digitata* colonies are digitated to arborescent with irregular, anastomosing branches. The branches become thinner, anastomosing, terete, or tapering from colonies in protected subtidal biotopes. *Montipora digitata* has a morphology similar to *M. samarensis* and *M. altasepta*, which can cause some difficulty in identifying them using only traditional morphological methods; thus, more up-to-date classification and molecular methods are urgently needed for coral identification.

*Montipora digitata* samples (TIOSOA-LCC-2013-Monti. Dig.) were collected in the Laboratory of Coral Conservation, Third Institute of Oceanography, SOA. *Montipora digitata* colony was obtained in 2013 from Houhai coral reefs in Sanya, Hainan Province. We used next generation sequencing (NGS) to perform low-coverage whole-genome sequencing according to our previous protocol (Shen et al. 2014). Initially, the raw NGS reads were generated from HiSeq X Ten (Illumina, San Diego, CA). About 0.09% of the raw reads (31,475 out of 33,430,528) were assembled *de novo* by using commercial software (Geneious V9, Auckland, New Zealand) in order to produce a single, circular form of a complete mitogenome with about an average 264× coverage.

The complete *M. digitata* mitogenome was 17,884 bp in size (GenBank KY094489), and its overall base composition was 24.9%, 14.2%, 24.2%, and 36.8% for A,C,G, and T, respectively, with a GC content of 38.3%. It showed a 99% identity to *Anacropora matthai* (GenBank AY903295) after the BLAST search against NCBI nr/nt database. rRNA and tRNA protein coding *M. digitata* mitogenome genes were predicted using DOGMA (Wyman et al. 2004), ARWEN (Laslett and Canback 2008), and MITOS (Bernt et al. 2013) tools followed by manual inspection. The complete mitogenome of *M. digitata* includes 13 unique protein-coding genes (PCGs), two ribosomal RNA (rrnL and rrnS) genes, and only three transfer RNA (trnW, trnR, and trnM) genes. All PCGs and tRNA and rRNA genes were encoded on the H-strand except for trnR, which is encoded on the L-strand. It is interesting to note one big intron (11,489 bp) is inserted in the ND5 gene.

To validate the phylogenetic position of *M. digitata*, we used MEGA6 software (Tamura et al. 2013) to construct a maximum likelihood tree (with 500 bootstrap replicates and Kimura 2-parameter model) containing the complete mitogenomes for 20 *Acroporidae* family-derived species. Corallimorphidae-derived *Corallimorphus profundus* was used as an outgroup for tree rooting. Result shows *M. digitata* is close to *M. cactus* with high supported bootstrap value (Figure 1). In conclusion, the complete *M. digitata* mitogenome deduced in this study provides essential and important DNA molecular data for further stony coral phylogenetic and evolutionary analysis.

**Figure 1.**
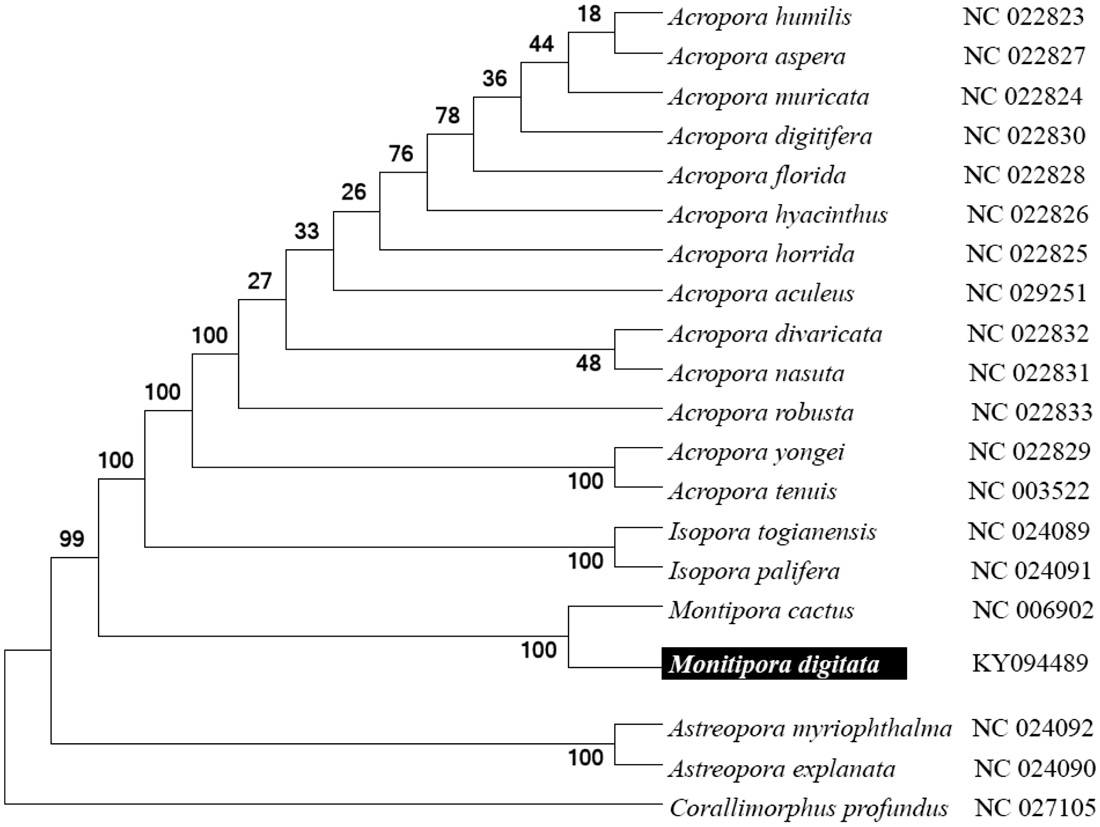
Molecular phylogeny of *M. digitata* and related species based on complete mitogenome. The complete mitogenomes are downloaded from GenBank and the phylogenic tree is constructed by maximum likelihood method with 100 bootstrap replicates. The gene's accession number for tree construction is listed behind the species name.
